# Robotic Surgery: A Comprehensive Review of the Literature and Current Trends

**DOI:** 10.7759/cureus.42370

**Published:** 2023-07-24

**Authors:** Yeisson Rivero-Moreno, Sophia Echevarria, Carlos Vidal-Valderrama, Luigi Pianetti, Jesus Cordova-Guilarte, Jhon Navarro-Gonzalez, Jessica Acevedo-Rodríguez, Gabriela Dorado-Avila, Luisa Osorio-Romero, Carmen Chavez-Campos, Katheryn Acero-Alvarracín

**Affiliations:** 1 General Surgery, Universidad de Oriente, Barcelona, VEN; 2 General Surgery, Universidad Mayor de San Simón, Cochabamba, BOL; 3 General Surgery, Universidad Autónoma de Baja California, Baja California, MEX; 4 General Surgery, Universidad Nacional del Litoral, Argentina, ARG; 5 General Surgery, Universidad del Zulia, Maracaibo, VEN; 6 General Surgery, Tecnológico de Monterrey, Monterrey, MEX; 7 General Surgery, Universidad Industrial de Santander, Colombia, COL; 8 General Surgery, Universidad Peruana de Ciencias Aplicadas, Lima, PER; 9 General Surgery, Universidad de Guayaquil Facultad de Ciencias Médicas, Guayaquil, ECU

**Keywords:** robot-enhanced surgery, minimally invasive surgical procedures, urology, robotic-assisted surgery, da vinci surgical system

## Abstract

Robotic surgery (RS) is an evolution of minimally invasive surgery that combines medical science, robotics, and engineering. The first robots approved by the Food and Drug Administration (FDA) were the Da Vinci Surgical System and the ZEUS Robotic Surgical System, which have been improving over time. Through the decades, the equipment applied to RS had undergone a wide transformation as a response to the development of new techniques and facilities for the assembly and implementation of the own. RS has revolutionized the field of urology, enabling surgeons to perform complex procedures with greater precision and accuracy, and many other surgical specialties such as gynecology, general surgery, otolaryngology, cardiothoracic surgery, and neurosurgery. Several benefits, such as a better approach to the surgical site, a three-dimensional image that improves depth perception, and smaller scars, enhance range of motion, allowing the surgeon to conduct more complicated surgical operations, and reduced postoperative complications have made robotic-assisted surgery an increasingly popular approach. However, some points like the cost of surgical procedures, equipment-instrument, and maintenance are important aspects to consider. Machine learning will likely have a role to play in surgical training shortly through "automated performance metrics," where algorithms observe and "learn" individual surgeons’ techniques, assess performance, and anticipate surgical outcomes with the potential to individualize surgical training and aid decision-making in real time.

## Introduction and background

Robotic surgery (RS) is an evolution of minimally invasive surgery that combines medical science, robotics, and engineering. Also known as robot-assisted surgery, it is a sophisticated technique that involves the use of specialized robotic platforms during surgical procedures to improve the precision of surgeons’ movements in complex procedures and small anatomical spaces. RS allows for the filtering of hand tremors, thereby improving flexibility and minimizing involuntary inaccuracies. As a result, it leads to fewer surgical complications such as surgical site infection, less pain, less blood loss, shorter hospital stay, quicker recovery, and smaller, less noticeable scars [1].

This system consists of two main components connected by data cables to a computer: the surgeon's master console and control devices [2,3]. The surgeon's master console is where the primary surgeon is located, and it provides a 3D dimensional view of the surgical field through an endoscopic camera inside the patient's body, which the surgeon can control from the console for a sense of immersion in the surgical field. Control devices such as handles or joysticks are used by the surgeon to perform surgical movements, which are then translated into real-time movements by the robotic arms located over the patient. These robotic arms have micro-joints that allow for the manipulation of surgical instruments and the endoscopic camera through laparoscopic ports connected to the patient's body [4].

The systems currently in use are not designed to act independently of human surgeons or to replace them. Instead, these machines act as fully controlled remote extensions of the surgeon and are best described as master-slave manipulators. The two main master-slave systems that have been approved by the United States Food and Drug Administration (FDA) and are in use are the da Vinci Surgical System (Intuitive Surgical, Mountain View, California) [5] and the ZEUS system (Computer Motion, Goleta, California) [6].

## Review

Evolution of robotic surgery

Robotic application in surgery has played a pivotal role in the remarkable advancement of medical technology in recent decades. The concept of RS originated from research in robotics funded by NASA and the United States Defense Research Advanced Projects Agency during the 1970s [7]. The primary objective was to create a system that would enable surgical procedures to be remotely controlled, thereby replacing surgeons in hazardous or hard-to-reach environments such as battlefields and space aircraft [8]. In 1985, the first-ever surgical robot called the Programmable Universal Machine for Assembly 560 (PUMA 560) was used for a neurosurgical biopsy at Westinghouse Electric in Pittsburgh, Pennsylvania [9]. Subsequently, in 1988, Imperial College in London developed a robotic system known as ProBot to assist in transurethral prostatectomies, featuring four axes of movement, a high-speed rotating blade for resection, and a compact size suitable for prostatectomy procedures [10].

Computer Motion, founded in 1989, emerged as the leading supplier of surgical robots. Their robotic arm, Automated Endoscope System for Optimal Positioning (AESOP), received approval from the FDA in 1994, making it the first telepresence surgical robot. This AESOP system was later enhanced and transformed into the ZEUS Robotic Surgical System, which featured three remotely controlled arms [11]. Concurrently, in the early 1990s, Integrated Surgical Solutions, Inc. (Alpharetta, Georgia) and IBM Corp. (Armonk, NY) collaborated to develop ROBODOC®, which successfully prepared the femur for hip replacement in human subjects in 1992 [10]. In 1999, Intuitive Surgical introduced the first da Vinci "Standard" surgical robot, which was used for the first time in 1998 at the Cleveland Clinic (Cleveland, Ohio).

Despite some limitations, such as the large size of the robotic arms and potential arm collisions, the da Vinci system gained acceptance in clinical practice. After a merger between Computer Motion and Intuitive Surgical in 2003, the da Vinci Surgical Systems became the dominant force in robotic-assisted laparoscopic abdominal operations, leading to the discontinuation of the ZEUS Robotic Surgical System. Since the FDA's approval of Intuitive Surgical's da Vinci Standard System in 2000, three newer generations of da Vinci Surgical Systems with increasingly advanced features have been developed: the S System (2003), the Si System (2009), and the Xi System (2014) [12].

The first da Vinci robot in 2000 had three arms with an endoscope attached to one and two instruments. Two years later, a four-arm robotic version was approved, providing improved exposure of anatomical structures and reducing reliance on a surgical assistant. The console had two handles controlled by the surgeon, eliminating hand tremors and scaling down movements for greater precision. The 2006 da Vinci S platform had a 3D HD camera and a touchscreen display. In 2009, the da Vinci Si model was released, allowing dual console surgery and improved training for non-expert surgeons. The Si robot had an upgraded image system and real-time fluorescence imaging. In 2011, platform adjustments allowed for single-port access. The most advanced system from Intuitive Surgical to date is the da Vinci Xi platform, which was released in 2014 [13].

Types of robotic surgical equipment and parts

The basis of most of the robots employed is the arms and the console. One arm has a camera, and the other has the surgical instruments attached, while the console gives a specific, magnified, high-quality view of the surgical site to the operator. The surgeon is leading other team members during the operation to assist him [14].

The Da Vinci Surgical System

The da Vinci Surgical System consists of three main structures, which are described as follows. (1) Surgeon cart: Through this instrument, the surgeon has a high-definition 3D view and access to the surgical site. (2) Patient cart: This is located alongside the patient’s bed, where we can find the camera and operative instruments controlled by the surgeon during the procedure. (3) Vision cart: This device makes a bridge between components to reach the high-quality image from the vision system. The da Vinci system could be differentiated into four different models to perform a minimally invasive surgery: the da Vinci Si, X, Xi, and SP. It is important to clarify that between all of them, the da Vinci SP model is implemented for single-port urological procedures, lateral oropharynx ectomy procedures (or radical tonsillectomy), and tongue base resection [15,16]. Other robotic systems have specific components depending on the specialty in which they are used, for example, the ExcelsiusGPS® is a computer-assisted RS equipment that is mainly used in neurosurgical and spine procedures. Its functioning is based on real-time navigation feedback through three imaging systems: intraoperative CT scan (3D), preoperative CT scan (3D), and fluoroscopy (2D) [16,17].

Versius Minimally Invasive Robotic System

Cambridge Medical Robotics Surgical (Cambridge, England), a UK company founded in 2014, developed Versius, a robotic system that aims to allow robotic minimally invasive operations for many surgical procedures including gynecological, urological, and colorectal surgical procedures. It is modularly conceived and consists of multiple identical arms, each mounted on a single support and taking small laparoscopic instruments (5 mm diameter). Each arm is very dexterous having seven degrees of freedom (DoF). The master console includes 3D‐HD imaging from the endoscopic camera with joystick controllers, and haptic feedback is available [18].

The Corindus CorPath GRX

This is a robotic system employed for robotic-assisted coronary interventions. It decreases radiation and lead exposure in surgical procedures and prevents potential surgical errors during the ones [16,19].

The Hugo RAS Robotic System

It consists of multiple parts to follow up. (1) Arm cart: Being variable for each procedure, it consists of a three- to four-arm configuration in robotic-assisted surgery and a one-arm configuration to assist with laparoscopic procedures. (2) Surgeon console: Besides the 3D high-quality vision, it is also compatible with its task simulator system. (3) Task simulator: It is developed for learning and practicing skills. (4) Tower: It consists of the central processing unit of the robotic and laparoscopic system [20].

There are several surgical robots actually in the market that have specific uses in each field, e.g., MSR‐5000 REVO‐I (thoracic urology), ALF‐X (thoracic, urology, and gynecology), Hintori (thoracic and urologic), Symani (open surgery), Avatera (thoracic, urologic, and gynecology), and Flex (transoral and urologic) [21].

ZEUS Robotic Surgical System

It was discontinued in 2003 with the development of the da Vinci device by Intuitive Surgical. Table 1 describes some differences that allow us to understand the evolution of surgical robots [6,21].

**Table 1:** Surgical Systems Robots ZEUS versus da Vinci features FDA: Food and Drug Administration; AESOP: Automated endoscopic system for optimal positioning.

	ZEUS	Da Vinci
Beginning	Started its production in 1995, and it was approved by the FDA in 2001.	Approved by FDA in 2011. Nowadays, it is the main reference in robotic surgery equipment.
Architecture	The ZEUS is a teleoperated system consisting of two robotic arms controlled by the surgeon through the AESOP and has a modular design.	The da Vinci is a teleoperated system with a control console from where the surgeon operates the robotic arms through four robotic arms placed in the patient’s trolley in a master-slave operating principle (one arm holds the camera, while the rest carries the surgical instruments). Motion is guaranteed by cable-driven joints at the distal end of the instrument.
Clinical use	The ZEUS was used in laparoscopic and thoracoscopic surgeries but was withdrawn from the market in 2003.	Da Vinci is widely used in various surgical specialties such as general surgery, urology, gynecology, and cardiac surgery, among others.
Cost	The ZEUS used to have a relatively lower cost compared to the da Vinci.	Da Vinci has a higher cost due to its more advanced technology and widespread clinical application.
Image generated	A standard screen that provides a 2D view of the surgical field or through polarized glasses with a different axis for each eye (adding the transmitted endoscopic image rapidly alternating on a screen equipped with an active polarizing matrix, reaching a 3D image).	Two cameras are associated with the master console that provides a magnified 3D view of the surgical field.

As for ZEUS RS, a lot of other systems have also been discontinued such as M7, Raven IV, PMAR, MiroSurge, Ottava, Bitrack, and MrBot.

Uses in urology

RS has revolutionized the field of urology, enabling surgeons to perform complex procedures with greater precision and accuracy. One of the primary uses of RS in this specialty is the robotic-assisted laparoscopic radical and partial prostatectomy for the treatment of prostate cancer. Also, it is used for the treatment of kidney cancer with robotic-assisted laparoscopic radical and partial nephrectomy and lymphadenectomy [22].

Robotic-assisted minimally invasive radical cystectomy, extracorporeal or intracorporeal urinary diversion, and ileal conduit are used for the treatment of bladder cancer. Its uses also involve the treatment of rectovesical and vesicovaginal fistula with single-port robotic-assisted urinary tract reconstruction and ureteral reimplantation as well as robotic-assisted biopsies. Furthermore, advanced techniques also include endoscopic inguinal lymphadenectomy (R-VEIL) for penile cancer, retroperitoneal lymph node dissection, creation of intracorporeal neobladder, robotic-assisted kidney transplantation, robotic-assisted pelvic floor reconstruction for females, robotic-assisted procedures for a variety of pediatric kidney disorders, and bladder and urethral reconstructive techniques for congenital malformations (e.g., urogenital sinus, prostatic utricle, among others) [23].

Uses beyond urology

RS has been widely adopted in various fields of medicine beyond urology. It was initially used in neurosurgery in 1985, primarily for biopsies [24]. Given its anatomical limitations, this technology has paused and advanced in different fields other than neurosurgery throughout the years. Its design depends on its specific application from the brain, spine, and peripheral nerves. Numerous neurosurgical operations can be performed with this technology, such as stereotactic biopsies, stereoelectroencephalography (sEEG), transoral odontoidectomy, spinal pathologies, neuroendovascular, and neuro-oncology treatments [25]. Nonetheless, many significant robotic technological milestones are anticipated to be overtaken in the upcoming years.

In the early 1990s, total hip arthroplasty was the first robotic orthopedic surgery application, followed by knee arthroplasty [26,27]. The spine was thereafter repaired by pedicle screw implantation, which is also one of the most popular surgical procedures in orthopedic RS [27]. With a drastic increase since 2014, studies have shown that RS increases alignment, limb lengthening, and patient satisfaction. However, despite financial and clinical barriers, many surgeons in the United States continue to perform only traditional, non-robot-assisted orthopedic surgery [28]. Interestingly enough, fracture fixation appears to have the potential for future advances in trauma surgery [29].

Since the 2000s, gynecology has applied this technique for common benign disorders, with hysterectomy and myomectomy being the most popular [30]. Other procedures include tubal reanastomosis to restore fertility, sacrocolpopexy, transabdominal cerclage, complex endometriosis surgery, as well as colpotomy and vaginal cuff closure [30,31]. Due to a lack of studies, advancements in malignant approaches continue to be explored. The excision of lymph nodes and surgical staging are currently used as diagnostic strategies for endometrial, ovarian, and cervical cancer [32].

In otolaryngology (ear, nose, and throat [ENT]), robotic-assisted surgery has been used to treat both benign and malignant pathologies as well. Depending on where the disease is located, interventions can fall into two categories: retroauricular hairline incision (RAHI) and transoral robotic surgery (TORS) [33]. Tonsillectomies, adenoidectomies, laryngectomies, and tumor removal in the head and neck area are examples of procedures that are currently active. TORS has also been used as an alternate treatment for obstructive sleep apnea, while it is not currently recommended [34].

Since the 2000s, there has been an increase in robotic cardiac surgery, with the majority of cases involving endoscopic coronary artery bypass grafting (CABG) and mitral valve repair (MVP) and relatively few involving aortic valve repair [35]. The first surgeries in the field of robotic cardiology were atrial septal defect (ASD) closures [36]. With the advancement in the field, although less common, patent ductus arteriosus (PDA) closure, intra-cardiac tumor removal, left ventricular lead placement, and atrial fibrillation (AF) surgery have taken place but are still in their infancy. However, due to a paucity of data, traditional cardiac surgeries remain favorable [35,36]. On the other hand, robotic thoracic surgery possesses the potential to become the standard of care for lung cancer lobectomy and mediastinal surgeries [37].

General surgery to RS has had very modest growth compared to other specialties despite its FDA approval in 2001. Certain factors that contribute to the machine’s limitations on certain procedures are a lack of instrumentation and quadrant restrictions [38]. Foregut surgery, hernia repair, hepatobiliary, endocrine, colorectal, and bariatric surgery are areas of practice today [39,40]. With more evidence and studies, rectal surgery is growing in popularity due to its oncologic safety and recovery benefits. On the contrary, open surgery is still the preferred method for hernia repair [40].

Advantages and disadvantages of robotic surgery

The benefits that come from the application of RS are better visualization as the operating surgeon obtains a three-dimensional image that improves depth perception; camera motion is stable and easily controlled by voice-activated or manual master controls. Furthermore, as compared to standard laparoscopic tools, the manipulation of robotic arm instruments enhances the range of motion, allowing the surgeon to conduct more complicated surgical operations [1]. When compared to open and laparoscopic surgery, RS causes far less physical discomfort. Ergonomic training can aid in the relief and prevention of ergonomic strain during RS [41]. Access issues can be alleviated with a preoperative screening endoscopy, and operational time decreases with expertise [42]. When compared to patients who had open surgery, RS reduced the chance of readmission by half (52%) and revealed a 77% reduction in the prevalence of blood clots (deep vein thrombosis and pulmonary emboli) [43].

The risks of RS are related to the complications associated with the usage of a robot, the patient positioning, robot malfunction, and the performed surgery. The loss of touch feeling, along with the power of robotic arms, may result in technical mistakes, longer operating times, and steeper learning curves. Inadequate touch sensation input may lead to the application of excessive force while manipulating tissues, resulting in unintended tissue injury [44]. The possibility of nerve injury due to the position of the patient has been reported in RS [45]. When compared to laparoscopic procedures, robotic procedures have been associated with longer operational durations as well as ocular problems, cardiac difficulties, endotracheal tube displacement, and nerve damage [46]. Robot faults have been documented in the range of 0.4%-4.6% [47]. In a study on bariatric surgery, Rogula et al. found no clinical advantage of RS over the conventional laparoscopic gastric bypass and that robotic gastric bypass extended the operating time [48].

Extension of robotic surgery in the world

Since the da Vinci robot was developed 15 years ago, the ongoing technological innovation has led to the growth of RS, and in today’s globalized world, the extension of this technology is undeniable. American, European, and Asian authors have described techniques for the performance of many different robot-assisted surgeries, proving not only its facility but also its safety [49]. However, the extent to which RS has been established in developed world nations has been unparalleled in the situation in developing countries.

For example, the limited financial resources for innovation, low health budget, and lack of training programs are obstacles when implementing RS in third-world countries. Regardless of the limitations, robotics implementation in Latin America is growing. In 2019, the number of procedures varied a lot in the region, highlighting Brazil with more than 21,000 surgeries since the acquisition of the first robot in 2008. The average number of surgeries performed by robots was between 525 and 625 procedures per system in Brazil, Chile, and Argentina [50]. Similarly, the Caribbean faces many challenges in providing all-inclusive surgical services for its people, and the largest among these are financial constraints [51]. The statistical situation is not that different in the Middle East, where only 1% of the da Vinci® Surgical Systems is installed worldwide, including 19 in Saudi Arabia, six in Qatar, two each in Kuwait and Lebanon, three in the United Arab Emirates, and only one in Egypt. Nevertheless, through partnerships and a quality-driven program, the introduction of RS is growing. By September 2017, 4271 systems were installed worldwide, including 2770 (65%) in the United States, 719 (17%) in Europe, 561 (13%) in Asia, and 221 (5%) in the rest of the world [52]. The number has significantly increased according to Intuitive Surgical Inc., the manufacturer of the da Vinci system. Now more than 6,500 da Vinci Surgical Systems are installed in 67 countries, and more than 55,000 surgeons worldwide have been trained in the use of da Vinci systems [53].

Costs of robotic surgery

Several benefits like a better approach to the surgical site, smaller scars, and reduced postoperative complications have made robotic-assisted surgery a trendy technique; however, some points like the cost of surgical procedures, equipment-instrument, and maintenance are important aspects to consider [54].

Regardless of whether robotic-assisted surgery has been used in the last 23 years, this minimally invasive procedure cost still outweighs other surgical interventions such as laparoscopy. Not only the cost of the machine which lies between $1.5 and $2 million dollars but also further allied costs like service and repair as well as training staff has limited its use in certain fields [55].

Cardiothoracic Surgery

Robotic-assisted mitral valve repair is considered a costly procedure with an increase of $2,064 per case in comparison with other interventions such as minimally invasive mitral valve repair (mini-MVR). On the other hand, the high procedural cost and equipment maintenance are countervailed by decreased transfusion requirements with a rate of perioperative blood transfusions of 22%, a lower rate of postoperative infection, shorter hospital lengths of stay, and better quality of life as patients tend to return to work earlier [56].

Gastrointestinal Surgery

Bariatric surgery costs vary depending on the technique selected by the surgeon, for instance, Robotic Sleeve Gastrectomy (RSG) tends to be more expensive ($8018), while Laparoscopic Sleeve Gastrectomy (LSG) costs $6505. In addition, patients are conducive to having a higher length of stay (LOS) with the robotic approach, 1.65 ± 1.07 days for LSG and 1.77 ± 1.29 days for RSG (P < 0.001). However, postoperative complications, mortality, 30-day readmissions, and pain management are similar in both groups, which makes the RSG a less preferred technique. Robotic Roux-en-Y gastric bypass has a higher cost of $10,325 per case, while LSG costs $8564, but the incidence of postoperative GI hemorrhage was significantly higher in the laparoscopic procedure [57].

Robotic Colorectal Surgery

Robotic colorectal surgery has the shortest LOS and lowest mortality percentages despite being costly and having more extended operative duration than other procedures like laparoscopy, making this approach better preferred [58]. For instance, the cost of robotic-assisted colectomies is $12,399, and the cost of the laparoscopic approach is approximately $10,335 [59].

Gynecologic Surgery

Halliday et al. [60] reported the results of a Canadian cost-consequence analysis of robotic hysterectomy compared with open hysterectomy. The authors reported that the robotic group had statistically significantly longer surgical time (351 ± 51 minutes compared to 283 ± 63 minutes; P = 0.0001), less blood loss (106 ± 113 mL compared to 546 ± 570 mL; P < 0.0001), greater uterine volume (120 ± 91 mL compared to 89 ± 102 mL; P < 0.05), less opioid use (one day or less, 50% compared to 4% [P = 0.0026]; three days or longer, 0% compared to 67% [P = 0.0001]), shorter time to tolerance of full diet in days (1.2 ± 0.4 compared to 3.5 ± 1.9; P < 0.0001), shorter LOS (1.9 ± 0.9 days compared to 7.2 ± 5.3 days; P < 0.0001), and fewer minor complications (19% compared to 63%; P = 0.003). The costs of the robot were included in the analysis, but the costs of the equipment, maintenance, and supplies were offset by the shorter length of hospital stay so that total hospital costs were lower in the robotic group ($9,613 ± $1,089 compared to $11,764 ± $6,790), assuming that five robotic cases would be performed per week [60].

Urologic Surgery

Bolenz et al. reported that the proportion of nerve-sparing procedures was statistically significantly different between robotic (85%), laparoscopic (96%), and open (90%) methods (P < 0.001). Differences were also seen in lymphadenectomy rates (robotic 11%, laparoscopic 22%, open 100%; P < 0.001), blood transfusion rates (robotic 4.6%, laparoscopic 1.8%, open 21.0%; P = 0.001), median operating room time (robotic 235 minutes, laparoscopic 225 minutes, open 198 minutes; P < 0.001), and median length of hospital stay (robotic 1 day, laparoscopic 2 days, open 2 days; P < 0.0001). Simultaneously, the authors reported statistically significantly different median direct costs in robotic ($6,752), laparoscopic ($5,687), and open ($4,437) surgical methods (P < 0.0001) [61].

Training in robotic surgery

RS is a new development of this century that requires systematized and structured training. Most of the programs involve systematic progressions to observation, case assistance, acquisition of basic robotic skills in the dry and wet lab settings along with the achievement of individual and team-based non-technical skills, and modular console training under supervision to an independent practice [62].

The basic training follows two phases: pre-console training (patient-side training) and console training. In patient-side training, the trainee must acquire experience in three areas: understanding the robotic system, bedside assistance, and basic laparoscopic skills [63]. These areas are explained in Table 2 [62,63].

**Table 2:** Patient-side phase for robotic surgical training

Patient-side training	Concepts involved
Basic robotic	Understanding of the robotic system components: console settings, secondary console, remote manipulator, arms, visualization support system, robotic accessories, cables, and connector. Learn troubleshooting for common errors.
Bedside training (bedside assistant)	Learn how the surgeon conducts the operation. Understand how the robotic arms interact with the patient. Troubleshoot errors that interfere with the surgery.
Patient position and port placement	Learn the correct access to the patient to have an adequate spatial triangulation between the robot patient cart and the organ. Correct port placement and patient position prevent complications such as neurologic, musculoskeletal, and hemodynamic issues. Familiarize yourself with the position variations of the bedside team (bedside assistant, scrub nurse, and anesthesia team).
Basic laparoscopic skills	Learn laparoscopic access, creation of pneumoperitoneum, removal of adhesions, application of clips, suction, retraction, and hemodynamic changes during laparoscopic surgeries.

The second phase starts with console training, which is based on acquired cognitive and technical skills to operate at the console. Most surgical programs transition to theses in the latter one to two years of residency. Prior to console training, the trainee must complete online modules with basic concepts available on the da Vinci surgery community website. The trainee will then be familiarized with camera and pedal skills, finger control with dry lab or virtual reality-simulated environment, and advanced console skills such as excision, suturing, and use of diathermy. Five virtual reality simulators such as robotic Surgical Simulator (RoSS™; Simulated Surgical Systems, Buffalo, NY), dV-Trainer™ (Mimic Technologies Inc., Seattle, WA), SEP Robot™ (SimSurgery™, Norway), the da Vinci Skills Simulator (Intuitive Surgical, Sunnyvale, CA), and RobotiX Mentor™ (3D systems, formerly Symbionix, Israel [62] exist.

Also, the virtual reality simulators have procedure-specific components that help to train to mimic and register procedure-specific movements; some of the examples are Hands-On Surgical Training (HOST™) in the RoSS™ and maestro AR in the dV-Trainer™ [62].

The RoSS Platform

It shows a realistic surgeon console with a 3D display, master controllers, and foot pedals that are programmed with 16 modules including Fundamental Skills in Robotic Surgery (FSRS) modules and proprietary HoST modules. The tasks consist of instrument and camera control, fourth arm control, ball placement, spatial control, needle handling, basic electrocautery, tissue cautery, tissue retraction, blunt tissue dissection, vessel dissection, and knot tying. All are evaluated in four modules, in which the tasks have three levels of difficulty with the respective evaluation stage [64].

The dV-Trainer

It has more than 60 training exercises, has both basic and advanced skills, familiarizes the trainee with operating the surgeon console, and teaches skills such as needle control, knot tying, energy application, and dissection. Procedure-specific training uses virtual instruments and augmented 3D case videos to improve clinical decision-making and procedural knowledge. Team training modules are also available to allow the surgeon and first assistant to train together [64].

The Da Vinci Skills Simulator (dVSS, Intuitive Surgical)

It has training for basic surgical skills. The user trains with the same robotic console used in actual surgical procedures, facilitating familiarization with the system. It also features user performance tracking, providing a means to measure user skill improvement over time [64].

The RobotiX: Mentor

It allows to personalize the specific curriculum of each trainee and has modules on robotic basic skills, fundamentals of robotic surgery (FRS), robotic essential skills, wristed and single site suturing, stapling, prostatectomy, hysterectomy, hysterectomy procedural tasks, vaginal cuff closure, lobectomy, and inguinal hernia repair [64].

The practice in the dry lab can be done in live or cadaveric animals or human models. The simulation has real-time procedures that help to learn troubleshooting in camera and clutch control, the position of hands, routine beads, needles, sutures, or vascular and bowel models. The wet lab can be practiced in frozen animal-human parts and live animals. This lab allows the trainee to acquire experience in handling the consistency and reaction of tissues to instrument touch as well as the use of diathermy and vascular control [62].

Once the trainee completes this phase, he or she will start training in the operating room to learn the steps of procedures from a mentor by familiarizing with specific procedures through reading and case observation with each step of every procedure, followed by bedside experience and console operating time [63].

The most widely used and well-supported assessment tool to evaluate the assessments of a surgeon's skills during RS has been the Global Evaluative Assessment of Robotic Skills (GEARS), which asks to rate the surgeon's performance on a scale from 1 to 5 in six domains: depth perception, bimanual dexterity, efficiency, force sensitivity, autonomy, and robotic control [64].

Latest developments and upcoming trends

This is an era of RS where a robot could either perform preprogrammed tasks or learn from its own experience through a feedback pipeline of good and not-so-good outcomes, driven by deep-learning models (DLM). Artificial neural networks (ANN) are the digital equivalent of the biological nervous system. DLM built with ANN is the intermediate stage for building autonomous robots. Currently, some tools needed to make independent robots are 2D surgical scene segmentation, depth-chart reconstruction, surgical skill evaluation, and surgical simulation and planning. Artificial intelligence uses algorithms to give machines mortal-like capacities to make opinions and perform cognitive functions, and it may well represent the future of surgical robotics [65].

Machine learning will likely have a role to play in surgical training in the near future through "automated performance metrics," where algorithms observe and "learn" individual surgeons’ techniques, assess performance, and anticipate surgical outcomes with the potential to individualize surgical training and aid decision-making in real-time [66].

There is no proof currently that AI can identify the critical tasks of robotic-assisted surgery operations, which determine patient outcomes. There is an urgent need for studies on large datasets and external validation of the AI algorithms used [67].

Increasing the amount of autonomy in RS surgical systems has the potential to standardize surgical outcomes that are independent of surgeons' training, experience, and day-to-day performance changes. The survival study results indicated that the developed robotic system could match the performance of expert surgeons in metrics including leak-free anastomosis and lumen patency and at the same time could exhibit an elevated level of consistency [68].

RS in the emergency setting has not been explored, although some early experience has been reported in the literature [40].

## Conclusions

In conclusion, RS has emerged as a significant advancement in the medical landscape combining medical science, robotics, and engineering to enhance surgical procedures. The use of specialized robotic platforms during surgery improves precision and flexibility and minimizes complications such as infections, pain, and blood loss. Over the years, RS has evolved and found applications beyond urology, including neurosurgery, orthopedics, gynecology, ENT, cardiac surgery, and general surgery. It has revolutionized procedures in these fields, enabling surgeons to perform complex operations with greater accuracy and patient satisfaction. Robotic surgical equipment and systems vary in design and components, tailored to specific surgical specialties. The advancement of robotic technology has led to the development of various models and features, such as 3D imaging, touchscreen displays, real-time navigation feedback, and haptic feedback, among others.

There are also limitations and challenges to its widespread adoption such as financial barriers as well as the need for further studies and evidence in certain surgical subspecialties. However, as technology continues to advance, RS is expected to play an increasingly prominent role, paving the way for future advancements and improvements in patient care.
